# Exploring biocultural diversity: A systematic analysis and refined classification to inform decisions on conservation and sustainability

**DOI:** 10.1007/s13280-025-02168-y

**Published:** 2025-04-16

**Authors:** Irene Otamendi-Urroz, Cristina Quintas-Soriano, Jan Hanspach, Juan Miguel Requena-Mullor, Anna Sophie Lagies, Antonio J. Castro

**Affiliations:** 1 Social-Ecological Research Laboratory, Biology and Geology Department, Andalusian Center for Global Change - Hermelindo Castro (ENGLOBA), University of Almeria, Carretera Sacramento s/n, La Cañada de San Urbano, 04120 Almería, Spain; 2 FRACTAL Collective, Madrid, Spain; 3 Faculty of Sustainability, Leuphana University Lüneburg, Universitätsallee 1 C11.210d, 21335 Lüneburg, Germany

**Keywords:** Biocultural approaches, Biocultural diversity components, Cluster analysis, Conservation efforts, Evidence synthesis, Social-ecological systems

## Abstract

Biocultural diversity reflects the interplay among biological and cultural diversity within social-ecological systems, shaped by human societies’ unique interactions with nature. This concept encompasses diverse knowledge, beliefs, practices, and values linked to local biodiversity. Despite its growing relevance, research gaps persist, including the need for theoretical clarity and a broader empirical application. To examine biocultural diversity’s role in conservation and sustainability, we conducted a systematic mapping of scientific literature in English and Spanish from 1990 to 2021, using cluster analysis to identify research trends. Including Spanish literature broadened the analysis, offering unique perspectives often overlooked. Results reveal gaps on research, particularly regarding intangible biocultural components (e.g., traditional knowledge, values, or worldviews) and the need for research in underrepresented regions like Africa and Asia. To advance in this direction, we propose a novel classification for biocultural diversity to foster holistic understanding, encourage exploration of intangible components, and promote collaboration among stakeholders.

**Supplementary Information:**

The online version contains supplementary material available at 10.1007/s13280-025-02168-y.

## Introduction

During the last decades, global sustainability challenges including biodiversity loss, climate change, resource depletion, and social inequality, have grown more complex. This has led to the implementation of new approaches that holistically acknowledge the diversity of human-nature interactions and worldviews (Hanspach et al. 2020; Burke et al. 2023). These approaches aim to enhance human well-being while preserving nature, acknowledging the mutual influence between human cultures and ecosystems (Posey 1999; Rapport and Maffi 2010). Among these, biocultural approaches have gained significant attention in academia as a strategy for overcoming sustainability challenges and fostering conservation initiatives (Hanspach et al. 2020). Biocultural approaches have emerged as practical strategies and actions designed to conserve and operationalize the broader conceptual framework of biocultural diversity.

‘Biocultural diversity’ was first defined as the ‘diversity of life in all its manifestations—biological, cultural, and linguistic—which are interrelated and co-evolving within a complex socio-ecological adaptive system’ (Maffi 2005; 2007). Since this initial definition, the concept of ‘biocultural diversity’ has been defined and redefined multiple times, either treating linguistic diversity as a distinct category or embedding it within the cultural dimension (Bridgewater and Rotherham 2019).

For the purposes of this study, we adopt the definition proposed by Loh and Harmon (2005), which states that biocultural diversity includes biological diversity at all its levels, from genes to populations, species, and ecosystems; cultural diversity spanning from individual ideas to entire cultures; and all the interactions among them. Biocultural diversity recognizes that human societies have developed unique ways of interacting with and utilizing the natural world, resulting in a diversity of knowledge, beliefs, practices, and values that are intimately linked to the biodiversity of their local environment (Maffi 2005). It also captures the dynamics of ‘biocultural memory’, which refers to the collective knowledge, practices, beliefs, languages, and values transmitted across generations that inform and shape human interactions with the environment (Lindholm and Ekblom 2019). Globally, different cultures and peoples perceive, manage, and appreciate biodiversity in different ways because of their distinct heritage and experience (Cocks 2006). Therefore, biocultural diversity is context-specific and dynamic over time (Bridgewater and Rotherham 2019).

Despite its growing recognition in the academic world, biocultural diversity research faces a challenge in translating theoretical approaches into practical implementation (Vierikko et al. 2017; Bridgewater and Rotherham 2019; Lukawiecki et al. 2022; Zhao et al. 2022; Wall et al. 2023). In general, resources for research are limited (Vierikko et al. 2017; Zhao et al. 2022), and particularly, biocultural diversity research demands extensive datasets requiring substantial sampling efforts (Vierikko et al. 2017; Zhao et al. 2022). In addition, research and actions require the integration and application of practical interdisciplinary expertise and the collaboration between many different actors (Lukawiecki et al. 2022; Zhao et al. 2022). More importantly, the concept still requires theoretical clarification due to the lack of consensus in its definition, with multiple interpretations emphasizing different aspects of the biological-cultural relationship (Bridgewater and Rotherham 2019). Additionally, the term ‘biocultural’ is used differently across disciplines further complicating its conceptual coherence (e.g., in anthropology, it refers to the study of human biological evolution in relation to cultural influences, particularly in terms of nutrition and biological adaptation) (Franco 2022). This ambiguity makes it difficult to establish clear boundaries on what does and does not constitute biocultural diversity, increasing the risk of the concept being underutilized. Furthermore, biocultural diversity needs indicators for its quantification and the integration of practical applications into interdisciplinary frameworks (Gavin et al. 2015; Hanspach et al. 2020; Lukawiecki et al. 2022; Zhao et al. 2022; Burke et al. 2023; Wall et al. 2023; Díaz-Reviriego et al. 2024).

In order to advance the clarification of the concept, we conducted a systematic mapping to address the following question: ‘To what extent can incorporating lessons from prior empirical evidence and practical applications support the advancement and strengthening of biocultural diversity research?’. By doing so, we gathered information on how biocultural diversity has been studied and applied to address conservation and sustainability issues. We categorized the empirical evidence using a data-coding framework designed to explore the following secondary questions:

- Where has the concept of biocultural diversity been applied for facing environmental and sustainability issues?
- What components of biocultural diversity have been empirically studied?

Understanding these geographical distribution trends and biases toward specific biocultural diversity components is essential for identifying gaps in previous biocultural diversity research. From these gaps, future opportunities to learn could emerge, leading to the implementation of new empirical and practical applications that contribute to the progress of biocultural diversity research and conservation.

Recent systematic reviews on biocultural diversity have primarily focused on its relationship with sustainability (Hanspach et al. 2020; Díaz-Reviriego et al. 2024), traditional ecological knowledge (Sharifian et al. 2022; Burke et al. 2023), and pathways for conserving and enhancing it (Lukawiecki et al. 2022; Zhao et al. 2022). Nevertheless, to the best of our knowledge, there has not been a comprehensive global systematic mapping of biocultural diversity that simultaneously considers scientific literature written in English and Spanish. The integration of Spanish literature alongside English sources is one of our study key contributions. Much foundational literature on biocultural diversity (Rozzi et al. 2002; 2008; Boege and Chan 2008; Toledo and Barrera-Bassols 2008) has been published in Spanish, as it originated from Latin America, where the concept has been deeply explored due to the region’s high biocultural richness. Since English dominates scientific publishing, excluding Spanish sources would reinforce an Anglocentric bias and overlook key contributions from scholars working in some of the world’s most bioculturally diverse areas. While we acknowledge that valuable research exists in many other languages, practical limitations prevented us from including more languages. By including literature in these two languages (English and Spanish), we aimed to access a wider range of scholarly articles and ensure a more inclusive representation of biocultural diversity research using a holistic understanding of the concept (Otamendi-Urroz et al. 2023a).

Consequently, this paper aims to: (1) identify and assess the empirical evidence on biocultural diversity, (2) build a spatially explicit database of empirical evidence on biocultural diversity, and (3) integrate empirical evidence to inform future decision-making processes for biocultural diversity conservation and sustainability.

## Methods

### Systematic mapping process

We conducted the systematic map between July 2022 and May 2023 following the Otamendi-Urroz et al. (2023a)’s protocol (Appendix S1), and based on the Collaboration for Environmental Evidence Guidelines and Standards for Evidence Synthesis (2022) and the ‘RepOrting standards for Systematic Evidence Syntheses’ (ROSES) for systematic map protocols (Haddaway et al. 2018). All the comprehensive details necessary for replicating this systematic mapping process are available in this paper’s supporting information (Appendix S4, S5 and S6).

We combined in a database the scientific articles on biocultural diversity previously selected in the systematic reviews conducted by Hanspach et al. (2020), Burke et al. (2023) and Díaz-Reviriego et al. (2024). Hanspach et al. (2020) queried the Scopus database^1^ with the search strings ‘biocultural’ OR ‘bio-cultural’ in Titles, Keywords and Abstracts for publications between 1990 and 2018 in English (1359 articles). On the other hand, Burke et al. (2023) and Díaz-Reviriego et al. (2024) used the search string ‘biocultural’ OR ‘bioculturales’ in Titles, Keywords and Abstracts for articles written in Spanish and published between 1990 and 2018 (Burke et al. 2023) and from 1990 to 2021 (Díaz-Reviriego et al. 2024) in 4 different databases: ‘Scielo’,^2^ ‘Redib’,^3^ ‘Redalyc’^4^ and ‘Dialnet’,^5^ obtaining a total of 642 articles.

We complemented the aforementioned databases by searching in the Scopus database for English articles between 2019 and 2021 (478 articles) following the keywords and criteria established by Hanspach et al. (2020) (Appendix S6). We combined the articles from all bibliographic database search results into the Zotero reference management software to assemble our library of academic literature (Appendix S2 and S3).We conducted a two-step screening process for all the collected articles (Appendix S6). In the first step, we screened titles and abstracts for relevance. We included publications related to sustainability, environmental issues, and natural resource management, while excluding articles focusing solely on paleontology, theology, psychiatry, human evolutionary biology, and biological anthropology. We also excluded non-English or non-Spanish articles because a rigorous review requires full comprehension of academic texts, and we, as authors, were only proficient in these two languages. Additionally, we excluded books and book chapters (Appendix S2).

Articles that met our eligibility criteria during the initial screening (678 articles) underwent a secondary screening process, where the full text of the article was reviewed (Appendix S4). We included empirical articles (i.e., papers that present original research based on primary data obtained from direct observation or experimentation at a specific study area) that explicitly mentioned ‘biocultural diversity’ and showed an in-depth engagement with the topic in the main text. Finally, from the 255 articles that met our eligibility criteria after the secondary screening, we excluded 28 articles that did not provide sufficient information regarding the variables under analysis (Appendix S4). Thereby we included a total of 227 articles for further analysis (Appendix S5; Appendix S6). This methodology helped us to identify relevant studies and extract data pertaining to various dimensions of biocultural diversity.

Two authors conducted the screening process, crosschecked by a third author. To assess repeatability, 3 co-authors simultaneously reviewed a random subset of 20 articles and their agreement on inclusion/exclusion decisions was evaluated. Subsequently, we proceeded with data extraction and coding of variables. We implemented a structured data-coding framework (Appendix S4) in order to enable the systematic organization and categorization of data extracted from the literature. The extracted data included key categories such as authors’ names, title, journal, year of publication, DOI, first author’s affiliation, study area, components of biocultural diversity studied, and current and future conservation efforts for biocultural diversity conservation (Appendix S4). Furthermore, in order to obtain a thorough understanding of the existing literature, the review process incorporated both deductive and inductive approaches, as well as quantitative and qualitative ones (Hanspach et al. 2020).

The systems classification was derived from the anthromes classification proposed by Ellis et al. (2021) which was adjusted to account for distinctions among terrestrial, inland waters, and marine systems, as well as various human management types.

Our classification for biocultural diversity components builds upon Elands et al.’s (2019) framework, which delineates and defines three main dimensions of biocultural diversity: materialized, lived, and stewardship. One of the key reasons for selecting this framework was that it offered a fully integrative perspective on biocultural diversity. Instead of treating biological and cultural components as separate entities that occasionally interact, Elands et al. (2019) place the central emphasis on the dynamic interactions between these components, recognizing that it is through these interactions that biocultural diversity emerges. While their framework offers a valuable and comprehensive foundation for understanding the multidimensional nature of biocultural diversity, it offers examples of indicators (e.g., time spent in urban parks) primarily focused on urban contexts and lacks a detailed subcategorization within the main 3 biocultural diversity dimensions. This can make it challenging to systematically classify or identify specific biocultural diversity components in diverse research contexts.

To address this limitation, we proposed a refined classification that introduces subcategories within each of the three main dimensions identified by Elands et al. (2019). By creating a more detailed classification, our framework aims to facilitate the identification and categorization of biocultural diversity components across different disciplines and social-ecological contexts.

### Data analysis

We calculated relative frequencies for all coded variables. Subsequently, we employed a Principal Components Analysis (PCA) and a cluster analysis to explore the relationship among biocultural diversity components in R (Quintas-Soriano et al. 2019; R Core Team 2023). The PCA allowed us to reduce the dimensionality of our dataset and identify the main explanatory variables underlying the variability across articles. To determine the most influential variables, we selected the first six principal components, as they accounted for a high percentage of explained variance (> 90%) while minimizing redundancy. From the PCA results, we found that, among our initial set of 35 variables, six—materialized, praxis, corpus, language, kosmos, and stewardship—had the highest loadings on these six principal components, making them the most relevant in explaining the variability within the dataset.

To explore the similarities and dissimilarities between articles based on these 6 variables, we computed the Hamming distance (Hamming 1986) and constructed a dissimilarity matrix, essential for subsequent cluster analysis. The cluster analysis identified bundles of articles that studied similar combinations of biocultural diversity main components. We used the ward.D2’s linkage method to minimize the increase in the within-cluster variance as the criterion for merging clusters (Murtagh and Legendre 2014). We determined the optimal number of clusters using both graphical (i.e., by plotting within-cluster variance versus the number of clusters) (Kassambara and Mundt 2020) and numerical techniques (i.e., Scott index) (Scott and Symons 1971). Finally, we applied a non-metric multidimensional scaling (NMDS) for cluster visualization (Otamendi-Urroz et al. 2023b) by using the ‘metaMDS’ function implemented in the ‘vegan’ package in R.

### Methodological limitations

The findings of this systematic mapping must be considered with some limitations. While we analyzed articles in 2 widely spoken languages, English and Spanish, the scope of biocultural diversity is global. Although our mapping aimed to broaden the concept of biocultural diversity and minimize biases by considering these 2 languages (Lukawiecki et al. 2022), a more comprehensive global review requires the inclusion of additional languages. This inclusion is essential to ensure fairness to non-native English or Spanish-speaking researchers and to capture a more holistic view of the concept. Still, our findings provided a significant foundation serving as a starting point for future mappings that could encompass a wider array of languages.

We also acknowledge some limitations regarding the type of articles analyzed. By exclusively focusing on empirical scientific articles, our intention was to narrow our analysis to articles directly applying the biocultural diversity concept through scientific evidence derived from primary data obtained by direct observation or experimentation in specific study areas. Furthermore, the similar standardized structure of these empirical scientific articles facilitated the comparability across the different study cases.

However, this approach may have led to the exclusion of pertinent literature from other typologies of scientific literature such as reviews, discussions, methodological or conceptual articles and gray literature. While conceptual and review articles could offer valuable discussions on the definition and theoretical boundaries of biocultural diversity, our focus was on identifying how the concept is operationalized in empirical research—what aspects of biocultural diversity are studied, what components are emphasized, and how the concept is applied in real-world contexts. This approach helps to delineate what is commonly considered part of biocultural diversity, providing a foundation for a definition that aligns with empirical applications of the concept. Nonetheless, future systematic mapping efforts could benefit from broadening the search scope to include other types of publications (Lukawiecki et al. 2022).

## Results

### Studied areas

**Fig. 1:**
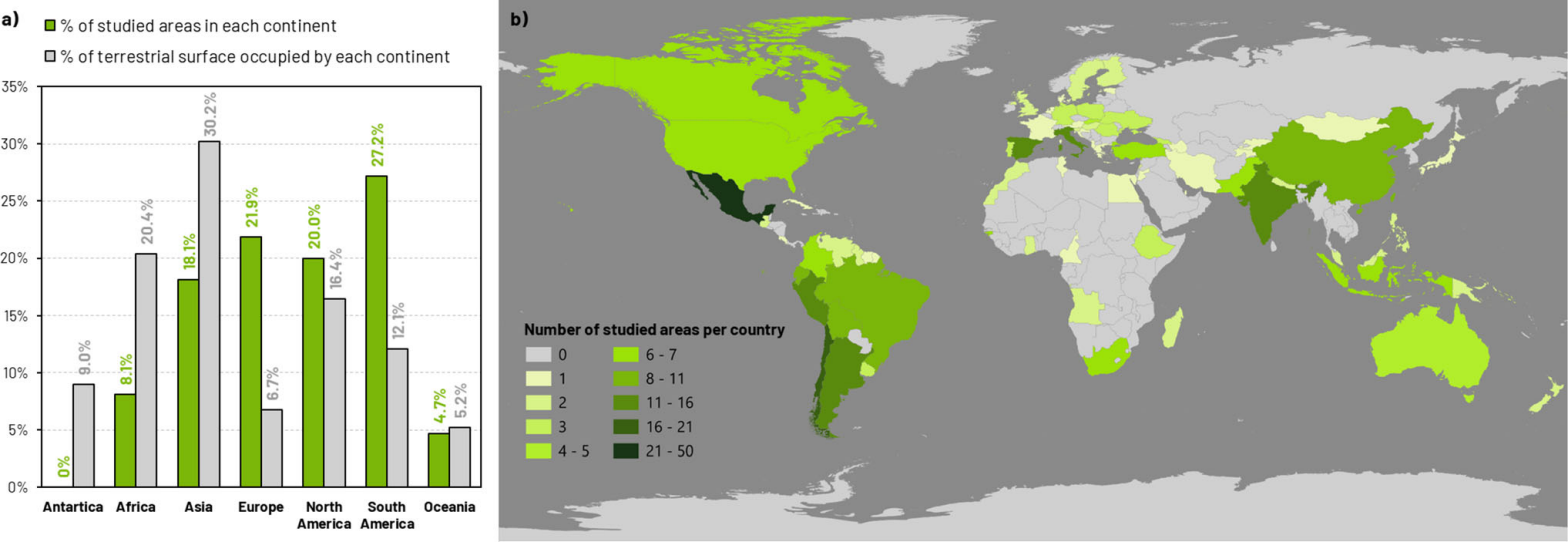
**a** Percentage of studied areas per continent (green) compared with the total percentage of land surface occupied by each continent (gray). **b** Number of study areas per country

The 227 articles analyzed covered a total of 323 different study areas. South America, Europe, and North America accounted for the majority of the study areas, while Africa and Asia remained understudied, despite being the continents that hold the most extensive terrestrial territories (Fig. 1a). Regarding countries, Mexico comprised the highest number of study areas (*N* = 47) followed by Chile (*N* = 18), Argentina (*N* = 14), India, Italy, Spain (*N* = 13, each), and Peru (*N* = 12) (Fig. 1b).

**Fig. 2:**
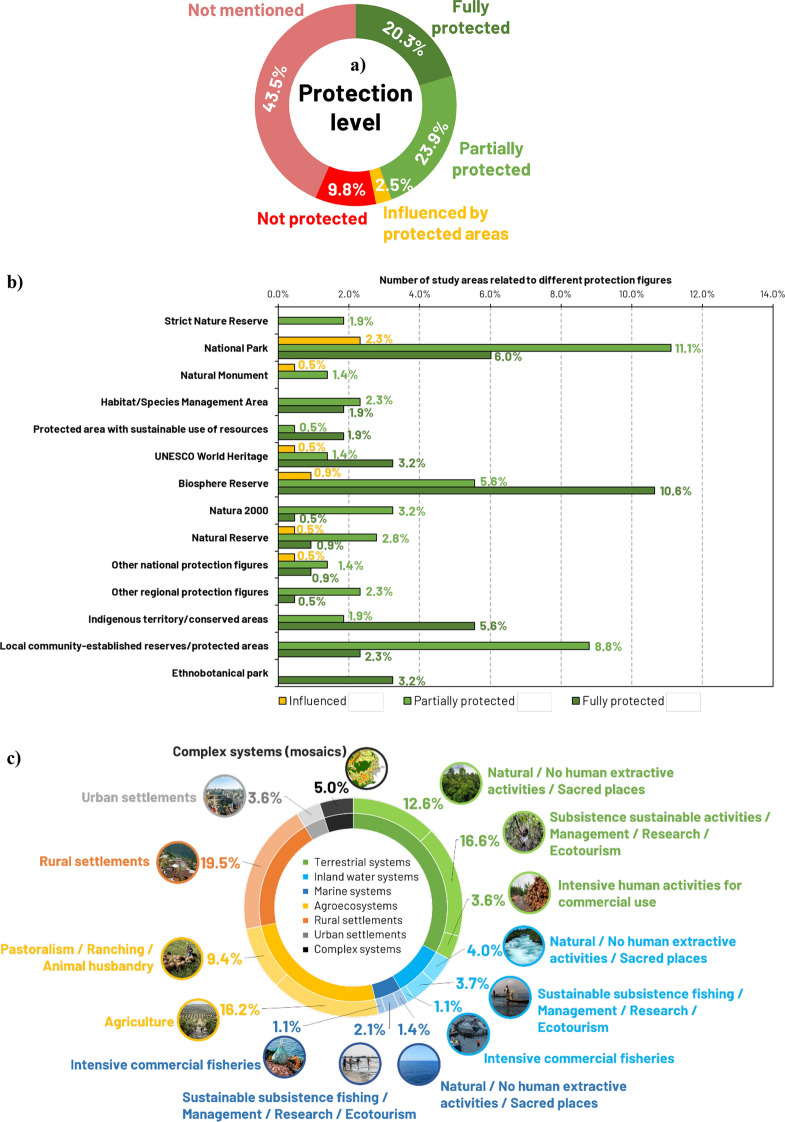
Study areas characterization. **a** Associated protection level, **b** predominant protection figures, and **c** types of systems studied

Approximately half of the study areas (46.7%) were fully or partially protected or influenced by different protection figures (Fig. 2a). The rest of the areas lacked protection (9.8%) or did not specify any protection figures (43.5%). National Parks were the most frequently represented form of protection figures (19.4%), followed by more integrative figures for conservation such as Biosphere Reserves (17.1%), and reserves established by local communities (11.1%) (Fig. 2b).

Different systems simultaneously occurred in the same study area. Terrestrial systems, agroecosystems, and rural systems were widely studied (32.8%, 25.6%, and 19.5% of study areas, respectively), whereas inland water, marine, and urban systems were understudied. We further categorized agroecosystems, terrestrial, marine, and inland water systems based on different human management types (Fig. 2c). The specific location of each study area and its characteristics can be interactively examined in the supporting information (Appendix S7).

### Biocultural diversity components

**Fig. 3:**
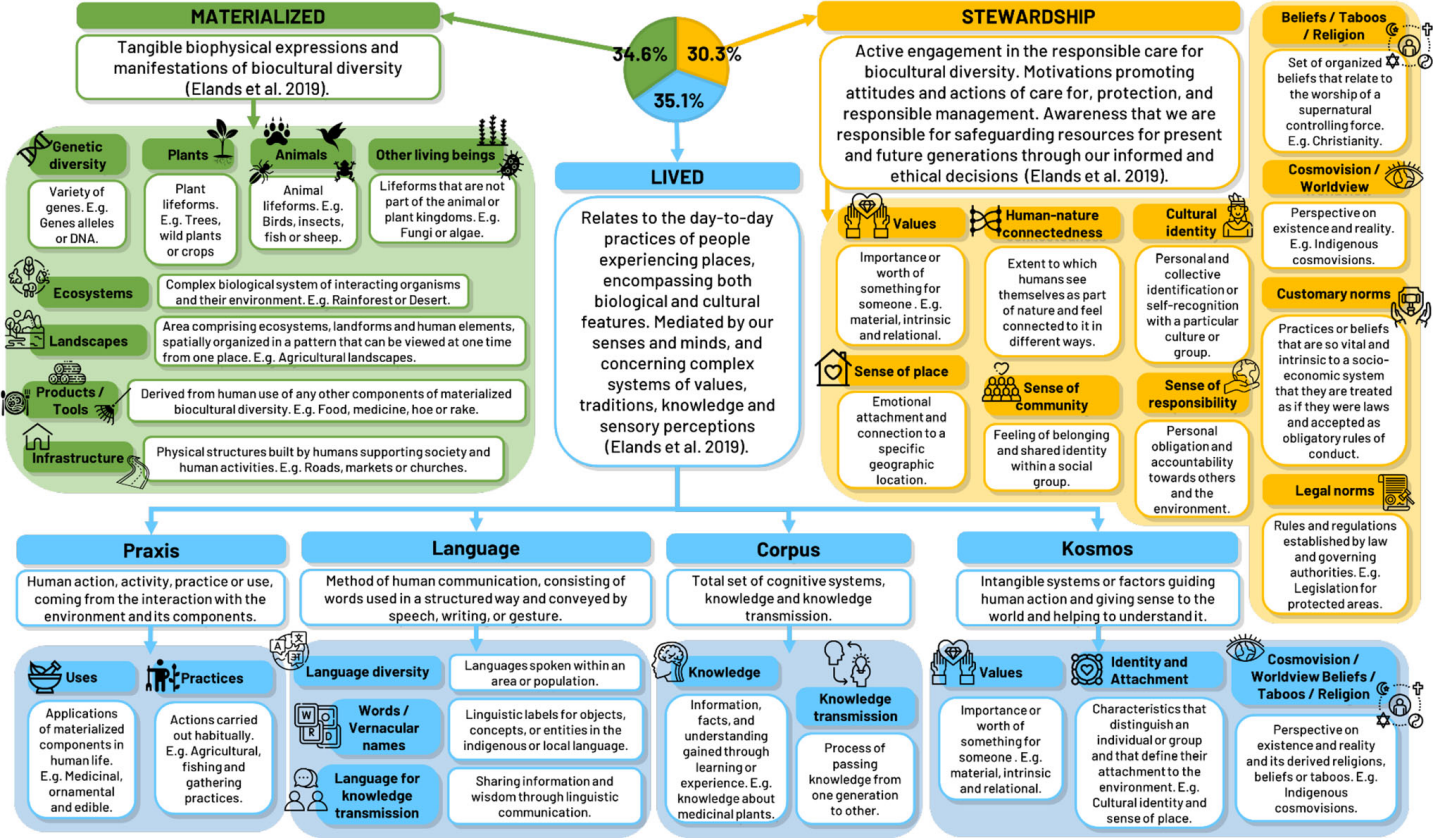
Biocultural diversity classification used for the study of the different components addressed by the reviewed research articles. Definitions and descriptions of components are provided within the boxes

Each dimension of biocultural diversity—materialized, lived, and stewardship—was fairly equally represented, with approximately 34.6%, 35.1%, and 30.3% of the studied components, respectively (Fig. 3).

In the context of the materialized dimension (i.e., tangible biophysical expressions and manifestations of biocultural diversity), we identified 8 different tangible components in our new classification. We divided the lived dimension (i.e., people’s daily practices mediated by the senses, traditions, knowledge, and values) into 4 new sub-dimensions (Fig. 3): (1) Praxis (2 components), which encompasses human practices and uses of materialized biocultural diversity (e.g., agriculture, transhumance, hunting or use of medicinal plants for home remedies); (2) Language (3 components), focusing on the diverse forms of human communication (e.g., names of plants and animals in indigenous language and their meanings); (3) Corpus (2 components), primarily addressing knowledge and its transmission (e.g., traditional ecological knowledge about medicinal plants transmitted orally in family gatherings); and (4) Kosmos (3 components), which involves intangible elements that provide meaning to human existence (e.g., relational values, sense of place, religious taboos or beliefs when visiting sacred places). Lastly, within the stewardship dimension (i.e., motivations promoting active engagement in the responsible care for biocultural diversity); we identified a total of 10 different components (e.g., legal norms, customary norms, values, worldviews or religion) for the new classification. Although stewardship is not always so evidently recognized as part of biocultural diversity, it plays a vital role in its long-term preservation by fostering ethical responsibility and practical commitments to its conservation. Moreover, it is worth noting that some components in the stewardship dimension and the kosmos sub-dimension overlapped. This overlap arises from the intricate nature of the intangible biocultural diversity components. These components can simultaneously be part of the lived dimension, experienced in people’s daily lives, and contribute to stewardship by encouraging a conscious commitment to the responsible care of biocultural diversity.

**Fig. 4:**
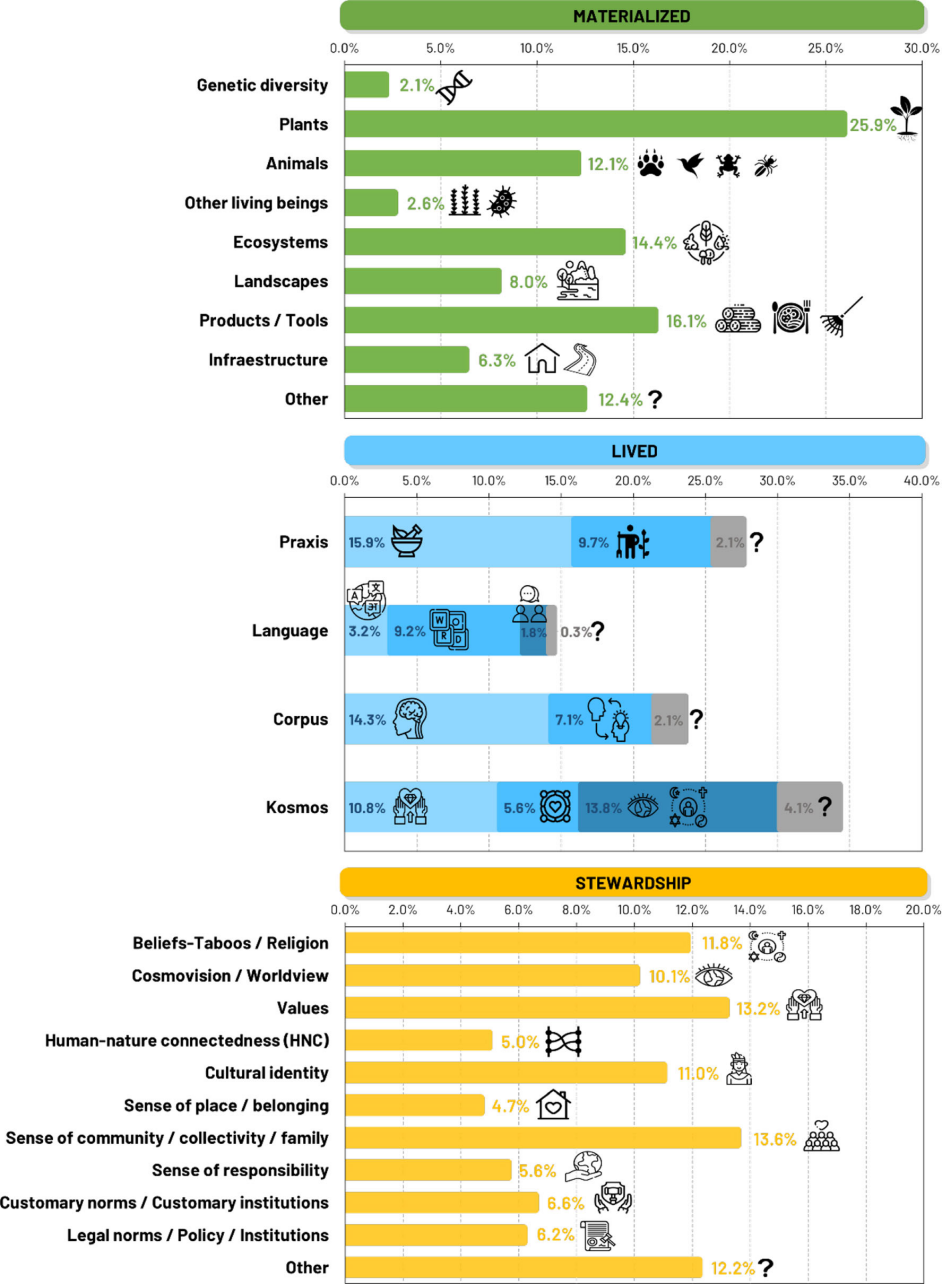
Percentages of each of the studied biocultural diversity components across the 3 main dimensions

The analysis revealed distinct emphases across biocultural diversity components (Fig. 4). In terms of materialized biocultural diversity, plant-related elements were the most studied (25.9% of materialized components), followed by products/tools (16.1%), and ecosystems (14.4%). Regarding lived biocultural diversity, the kosmos sub-dimension stood out as the most studied (34.3% of lived components) followed by praxis (27.7%), corpus (23.5%), and language (14.5%). Within the praxis sub-dimension, uses (15.9% of lived components) were the most represented, while within the kosmos sub-dimension, cosmovisions/worldviews (13.8%) held significance. Similarly, knowledge components took prominence (14.3%) regarding the corpus sub-dimension, and words/vernacular names (9.2%) within the language sub-dimension. Lastly, concerning stewardship biocultural diversity, the sense of community/collectivity/family (13.6%) and values (13.2%) were noteworthy.

### Cluster analysis and NMDS

**Fig. 5:**
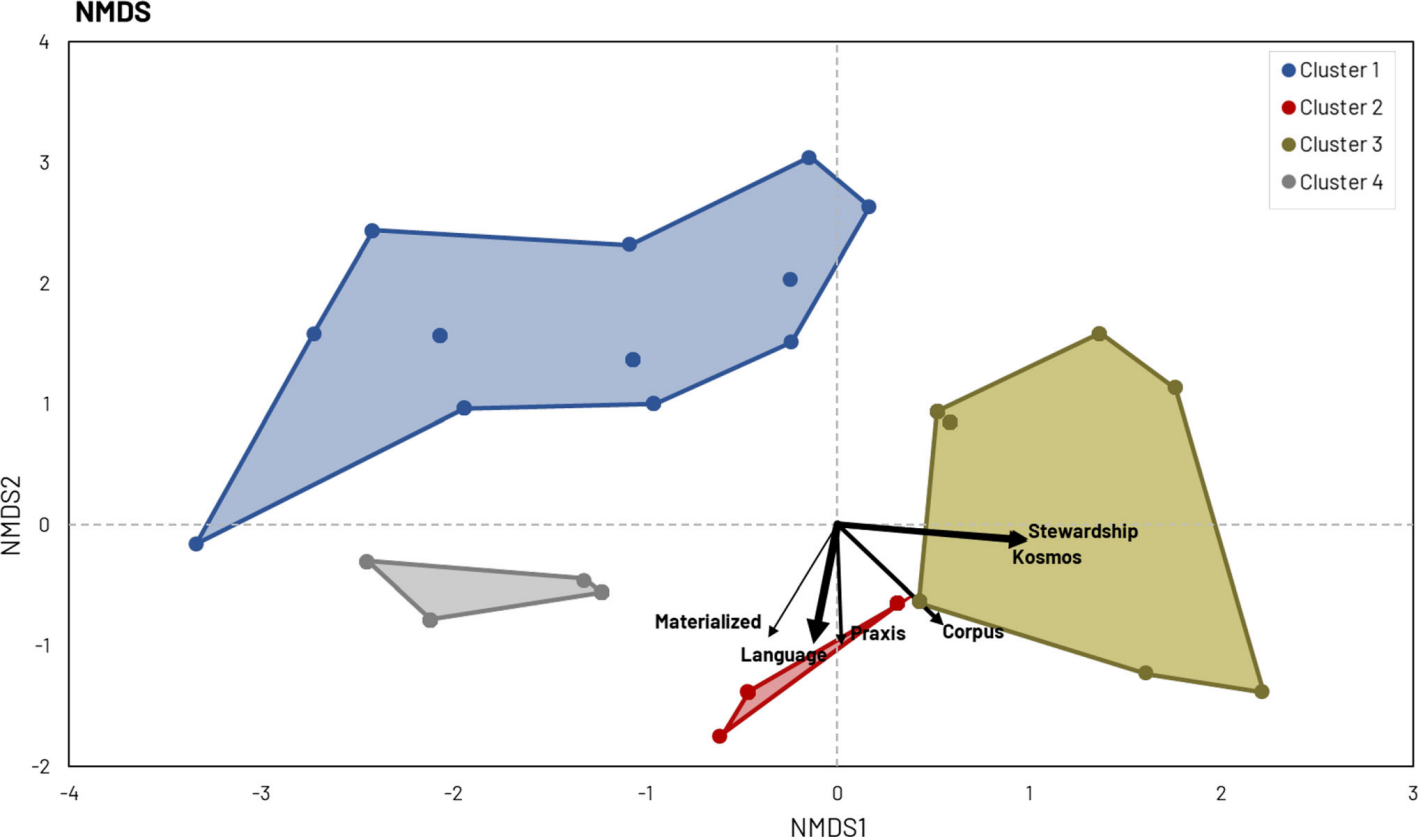
NMDS plot. Articles (data points) are organized into clusters (polygons) and positioned within a non-metric multidimensional scaling ordination space. Arrows represent the relationship between the direction and magnitude of change in the 6 biocultural diversity variables (components) and the ordination axes. The direction of the arrow indicates the direction of the steepest increase in the biocultural diversity variable. The arrow thickness indicates the correlation strength between the variable and the corresponding NMDS axis (the thicker the arrow, the higher the correlation). For example of articles in each cluster, see Fig. 6

**Fig. 6:**
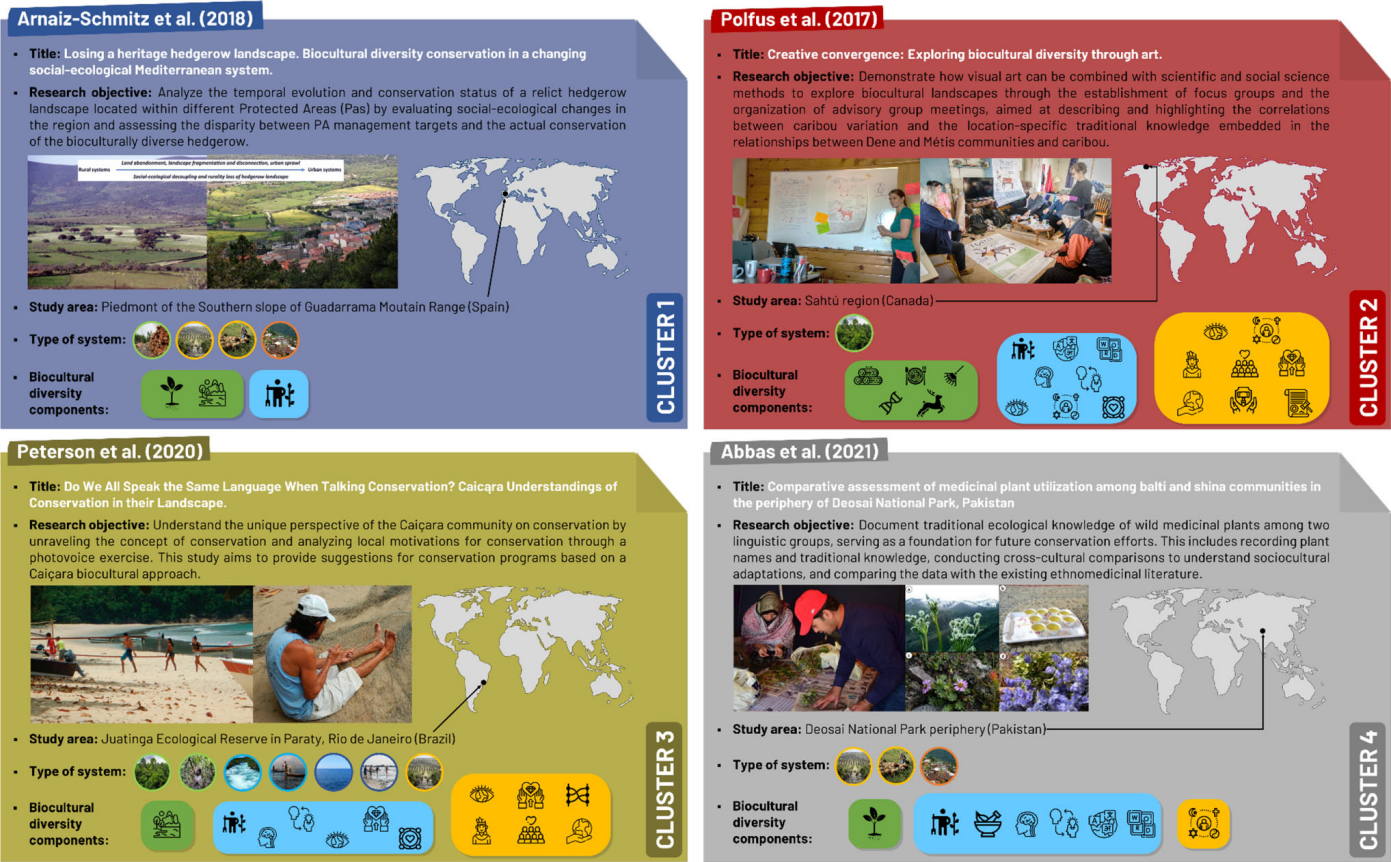
Flashcards with examples of articles within each cluster. Legends of the symbols representing the types of systems and the biocultural diversity components are provided in Figs. 2 and 3, respectively

The cluster analysis (Appendix S8; Appendix S9) identified 4 distinct groups of articles (*k* = 4) (Figs. 5 and 6) based on the components bundled from the biocultural diversity components classification (Fig. 3). Cluster 1 was the smallest group with 23 articles primarily focused on the materialized and lived dimensions of biocultural diversity. These articles concurrently explored fewer components, with an absence of language-related components. Additionally, the kosmos and stewardship dimensions received comparatively less attention within this cluster. Cluster 2 was the largest group with 101 articles that extensively examined various components of materialized, lived, and stewardship dimensions of biocultural diversity. Materialized, lived, praxis, corpus, and language components were prominently addressed in these articles, underscoring their holistic comprehensive approach to biocultural diversity. Cluster 3 (77 articles) explored a disparate number of components, with a predominant emphasis on the kosmos and stewardship components. However, when comparing with Cluster 2, language and corpus were relatively less relevant. Articles in Cluster 4 (26 articles) primarily targeted materialized, praxis, corpus, and language dimensions, often linked to ethnobotanical studies. It is worth mentioning that the kosmos and stewardship dimensions were not the central focus of these articles.

According to the association between the biocultural diversity components bundles and the NMDS axes (Fig. 5), we established a gradient of articles based on their comprehensive exploration and understanding of biocultural diversity. Articles within Cluster 2 emerged as the most comprehensive, thoroughly assessing a higher number of components in all three dimensions and adopting a holistic approach. We found Cluster 3, Cluster 4, and Cluster 1 in descending order of comprehensiveness. In Cluster 1, articles focused on a limited subset of components, providing a partial view of biocultural diversity rather than a holistic view (Fig. 6).

## Discussion

This study represents an in-depth effort to understand how biocultural diversity is studied globally. In the discussion below, we explore the global distribution of study areas in research articles, emphasizing disparities across continents and countries, and unraveling some of the motivations and criteria guiding researchers in the selection of study areas. Further, we delve into biocultural diversity components using a new classification, interpreting the dominance of certain dimensions and components, and arguing some of the possible reasons under the pattern observed across the 4 clusters of articles we found. The discussion calls for a balanced exploration, inclusive research, and collaborative efforts for sustainability and effective biocultural diversity conservation.

### Where has biocultural diversity been studied?

Our analysis covered a large number of case studies scattered across the world, but with a notable concentration of studies in South and North America. Some countries in Europe, southern and eastern Asia, and Oceania also emerged as key areas in which biocultural diversity has been studied. In contrast, most countries in Africa and western and northern Asia received comparatively less attention in research papers; despite these 2 continents encompassing the largest terrestrial surface areas and being widely recognized as important areas for biocultural diversity. We acknowledge that part of this imbalance might stem from the scope of our review, as it was conducted in a limited number of languages, potentially overlooking empirical studies published in other linguistic contexts.

Nevertheless, this geographic imbalance is not unique to biocultural diversity research but reflects broader structural inequalities that persist across multiple scientific disciplines. These disparities highlight the need for increased research efforts in underrepresented regions, particularly in Africa and Asia, where funding and academic opportunities remain limited (Petersen 2021). However, in pursuing these goals, several risks such as scientific colonialism, parachute science (i.e., scientists from Global North countries researching in Global South nations without adequately considering local interests or inhabitants) (Asase et al. 2021), or the Matthew effect (i.e., successful scientists from the Global North being more likely to receive increased research funding) (Bol et al. 2018) arise. Furthermore, while efforts to include local actors and knowledge in scientific studies have made progress, challenges remain. Even in biocultural diversity research, which is inherently expected to adopt a holistic and integrative approach, local stakeholders are often relegated to consultative roles rather than fully integrated into decision-making processes. This limits their direct influence on biocultural diversity research priorities and outcomes (Asase et al. 2021). These challenges are further exacerbated by the enduring legacies of colonialism, which continue to shape how biocultural diversity is studied and documented globally. In some cases, colonial histories have contributed to biocultural memory loss, particularly through displacement, language shifts (Mufwene 2002), and changes in land-use practices. However, in other cases, historical legacies have also shaped the ways in which biocultural diversity is conceptualized and recognized today (McAlvay et al. 2021; Zank et al. 2025).

To counteract these imbalances and avoid perpetuating scientific colonialism, funding agencies and institutions should prioritize direct financial support for researchers based in underrepresented regions, ensuring they have leadership roles rather than being secondary collaborators (Lukawiecki et al. 2022; Wall et al. 2023). Encouraging equitable partnerships through co-leadership models and long-term capacity-building programs can foster locally driven research rather than external interventions (OECD 2014). Additionally, greater investment in multilingual dissemination strategies would enhance the accessibility and visibility of research beyond dominant academic languages, promoting a more inclusive and representative global discourse. Addressing these structural barriers and expanding research efforts in underrepresented regions would enable biocultural diversity research to align more effectively with its principles of holism and inclusivity, paving the way for more impactful and context-relevant conservation outcomes.

At the country level, Mexico emerged as outstanding in terms of the number of studied areas. This prominence could be linked to Mexico’s status as one of the global biocultural diversity key areas, as noted in Loh and Harmon (2005). Moreover, it is important to note that the investigation in biocultural diversity originated and flourished, particularly in Latin American countries, with Mexico leading this trajectory, underscoring its historical importance and valorization through the lens of Indigenous peoples (Boege and Chan 2008; Toledo and Barrera-Bassols 2008). Recognizing this historical context underscores the vital contributions of Latin American scholarship in shaping the conceptual and empirical foundations of biocultural diversity. After Mexico, the most frequently studied countries were Chile, Argentina, India, Italy, Spain, Peru, Brazil and China. Some of them (India, Brazil, Peru) also form part of a Loh and Harmon’s (2005) list of biocultural diversity key areas and most of them corresponded again to Latin American countries with a long history of research related to biocultural diversity. Other countries such as Italy and Spain represent regions that also have a long studied and documented history of human-nature interactions and diverse management practices. Their geographical location in the Mediterranean basin, a biodiversity key area where the coevolution of human societies with the environment has unfolded over centuries, combined with socio-political contexts and research priorities, might be significant factors shaping the prevalence of biocultural diversity research (Martín-López et al. 2016; Dobrovodská et al. 2019; Quintas-Soriano et al. 2023). Beyond these regions, some countries have also emerged as key for biocultural diversity research in other continents that have been less studied (e.g., South Africa in Africa, Australia in Oceania, and Indonesia in Southeast Asia). These countries share a common feature: rich ecological and cultural landscapes intertwined with diverse Indigenous and local knowledge systems that contribute to the preservation and enhancement of biocultural diversity (Maffi and Woodley 2012).

In the light of these findings, we speculate that researchers might have selected study areas based on two primary criteria: (1) they targeted regions that initially exhibit high levels of biodiversity, ethnic-cultural diversity, and linguistic diversity (Maffi and Woodley 2012), which are currently recognized as key areas of biocultural diversity and typically concentrated between the tropics (Loh and Harmon 2005); and (2) they focused on areas with a long-standing human-nature relationship, allowing for the development of diverse management practices and activities over time. Regarding this second criterion, agroecosystems, rural settlements, and terrestrial systems (mainly forests) were the most extensively studied systems. These systems exhibit deep human-nature interactions via long-term management practices, mostly exemplified by agricultural practices and sustainable subsistence activities such as animal husbandry, beekeeping, and hunting. Notably, the selection of study areas by researchers might be influenced not only by these 2 criteria but also by the academic environment, scientific traditions, interests, funding, and political or cultural orientations (Bol et al. 2018; Asase et al. 2021).

Rural settlements, agroecosystems, and terrestrial systems encompassing traditional practices and sustainable subsistence activities have concentrated most attention in biocultural diversity research related to sustainability and environmental issues. In some particular cases, systems corresponding to these categories can be considered as potential ‘biocultural refugia’ safeguarded by local inhabitants and Indigenous peoples (Barthel et al. 2013; La Rosa et al. 2021; Ferrara et al. 2020; Quintas-Soriano et al. 2023). Biocultural refugia are places where relict species have found shelter during periods of stress, and that also contain a diversity of human knowledge, experiences, values, and belief systems (IPBES 2023). These refugia also serve as ‘libraries’ of traditional knowledge accumulated over time (Sõukand and Pieroni 2019; Ferrara et al. 2020; La Rosa et al. 2021), fostering resilience and offering historical solutions to a wide range of challenges, including current sustainability problems (Barthel et al. 2013; Hanspach et al. 2020; Díaz-Reviriego et al. 2024). Therefore, ‘biocultural refugia’ and ‘biocultural memory’ are intricately intertwined (Barthel et al. 2013; Lindholm and Ekblom 2019).

Our findings expand upon prior insights by emphasizing the role of biocultural refugia. We believe that biocultural refugia exist along a spectrum. At one end of the spectrum, we can find isolated ecosystems, such as remote mountainous regions or well-preserved natural rainforests with limited urban disturbance and external influences (e.g., Riozinho da Liberdade Reserve, Brazil) (Mooij et al. 2019), which serve as strongholds for endemic species, ancient cultural traditions, knowledge, and values. These areas represent the most traditional view of refugia as static, undisturbed environments. However, our analysis highlights the significance of more systems along this spectrum. Moving along the spectrum, we might encounter different types of rural ecosystems and agroecosystems with increasing external influences and pressures creating dynamic mosaics of habitats (e.g., rural landscapes between Guadarrama Mountainous Range and Madrid, Spain) (Arnaiz-Schmitz et al. 2018) and land uses (Cocks and Wiersum 2014) where the interaction between people and nature maintains biodiversity while providing humans with the goods and services needed for their livelihoods and well-being in a sustainable manner (i.e., production landscapes) (IPBES 2024). Finally, we can encounter rural communities situated really close to urban centers. These communities may face increasing pressures from urbanization, yet they still manage to uphold traditional practices, knowledge, and maintain connections to their cultural heritage (e.g., periurban homegardens near Buenos Aires, Argentina) (Castello et al. 2021). By positioning these diverse settings along a continuum, our findings provide a different perspective on how biocultural refugia adapt to and persist within varying socio-ecological contexts.

In addition, we observed that research on inland water, marine, and urban systems remains underexplored, probably due to the historical biases in research priorities favoring terrestrial systems. This may have shaped funding allocations, academic interests, and the overall distribution of attention, thereby influencing the research in favor of certain ecosystems at the expense of others. Challenges in researching aquatic environments, such as logistical complexities and limited visibility contribute to the neglect of inland water and marine systems. Moreover, urban systems, often seen as non-traditional settings with reduced human-nature interactions not considered as key areas, face additional obstacles due to high variability and rapid urbanization. Nevertheless, as cities expand and develop, investigating urban systems provides a unique opportunity to identify the causes of biocultural diversity loss in these areas, understand its dynamics at play, and develop strategies to mitigate and reverse these trends (Elands et al. 2019). In conclusion, our results underscore the need to expand biocultural diversity research to all these less-studied systems. However, addressing these gaps will require tailored methodologies that account for the logistical challenges of these environments.

Lastly, we found that nearly half of the study areas that may serve as shelters for biocultural diversity were protected or influenced by various protection figures (i.e., different types of protected areas, whether legally designated or established through customary and community-based governance systems). The protection of some study areas may arise from initial objectives that may not necessarily be focused on the preservation of biocultural diversity, but rather on biodiversity conservation. For instance, some study areas exhibit connections with well-established and historically prevalent protection figures (e.g., National Parks) that limit human activities and generate some conservation conflicts. Other study areas fall within Biosphere Reserves and local-community self-established reserves. Rather than being strict protection figures, these two come from a more emergent approach based on the integration of local inhabitants and their conservation practices within areas for its sustainable use. These governance models represent a shift from exclusionary conservation strategies toward more frameworks where human activities and ecological processes coexist, creating opportunities for biocultural diversity to flourish (Bridgewater 2002).

A reevaluation of conservation strategies is needed, advocating for a shift toward figures that actively engage and involve people in the conservation process while acknowledging their valuable traditional knowledge and practices (Bridgewater 2002; Elbakidze et al. 2013). Our findings underscore the potential for protected areas to evolve beyond static biodiversity reserves into dynamic spaces where biological and cultural diversity are actively maintained through collaborative management. An illustrative example of this approach is the Omora Ethnobotanical Park, located within the Cape Horn Biosphere Reserve in Chile. This initiative integrates scientific research, education, and conservation efforts with local cultural practices, demonstrating how biocultural diversity can be preserved through active community participation by incorporating indigenous and rural knowledge into ecological management (Rozzi et al. 2002; Rozzi et al. 2006; Rozzi et al. 2008; Rozzi et al. 2023; Tauro et al. 2021). Adopting this kind of biocultural approaches could help reconcile conservation conflicts by positioning local stakeholders as integral partners in decision-making processes. This shift would not only safeguard biocultural memory and traditional practices but also foster innovative solutions for managing socio-ecological systems. By reframing protected areas as hubs for co-management and collaboration, conservation efforts could more effectively address the challenges of biocultural diversity preservation and achieving sustainability (Bridgewater 2002).

### Elucidating biocultural diversity components

We proposed a new classification framework for biocultural diversity (Fig. 3) with the aim of contributing to the ongoing process of clarifying and operationalizing the concept (Bridgewater and Rotherham 2019; Lukawiecki et al. 2022; Wall et al. 2023). While biocultural diversity inherently embodies the interconnectedness between human societies and the natural world, influencing perceptions, practices, and relationships in multifaceted ways (Maffi 2005; 2007), we recognize the necessity of classification as a tool for enhancing comprehension and facilitating discourse surrounding biocultural diversity. Our classification does not seek to undermine the holistic nature of biocultural diversity, but rather to provide a structured framework that enables a more systematic and practical approach to studying and preserving its intricate connections.

Elands et al. (2019) elucidated 3 general dimensions of biocultural diversity (materialized, lived, and stewardship) and provided examples of some of their components in urban contexts. Building upon this foundation, we proposed a detailed classification of biocultural diversity components within each of these 3 dimensions. Our classification introduces sub-dimensions within the lived dimension (praxis, language, corpus, and kosmos) and details a broader range of components within each dimension. This not only enhances the systematic study of biocultural diversity but also addresses some of the challenges posed by the overlapping and intangible nature of certain components. For instance, some components can be part of the kosmos sub-dimension and the stewardship dimension at the same time. This overlap arises from the intricate nature of the intangible components. These components, such as values or sense of place, can simultaneously be part of the lived kosmos sub-dimension, as they are experienced in people’s daily lives, while also contributing to the stewardship dimension by encouraging a conscious commitment to the responsible care of biocultural diversity. Furthermore, our novel classification provides a comprehensive framework designed to be versatile and applicable across diverse settings, extending beyond urban areas. Nevertheless, this classification is open to discussion and improvement, particularly regarding the intricate overlap and relationships between components within the lived and stewardship dimensions.

The most studied components of biocultural diversity (i.e., plants, uses, knowledge, words/vernacular names, and the sense of community/collectivity/family) were related mostly to ethnobotanical studies, encompassing a high number of the articles analyzed. This result highlights the strong historical linkage between the concept of biocultural diversity and the field of ethnobiology. Ethnobiology was the first field to systematically approach and study biocultural diversity (Stepp et al. 2002). Historically, some biocultural diversity components have been addressed relatively straightforwardly through plant inventories (materialized dimension) and field surveys about plant uses (praxis) and plants’ vernacular names (language), all of them common methodologies in ethnobiological studies. On the contrary, the most abstract and intangible components of biocultural diversity from the stewardship dimension received limited attention or lacked in-depth exploration, due to methodological challenges. Our quantitative analysis revealed that there is a growing interest in biocultural diversity research for these intangible elements (e.g., cosmovisions, values, and worldviews) but coming from the perspective of the lived dimension. These results emphasized the need for a more balanced exploration of biocultural diversity components, particularly from the lens of the stewardship dimension, which often remains overlooked despite its critical role in fostering conservation actions and sustainability.

Despite the majority of analyzed articles following a holistic approach to biocultural diversity, we identified a gradient among the 4 clusters based on the comprehensive exploration and understanding of biocultural diversity. Articles ranged from a holistic approach, considering multiple and deep components simultaneously, to a more partial perspective on biocultural diversity, concentrating most of its attention on a few tangible components. For instance, Cluster 2 stood out as the most comprehensive, integrating components from all dimensions and sub-dimensions of biocultural diversity, which facilitated a deeper understanding of the complex systems and interactions studied. Meanwhile, Clusters 1 and 4 primarily focused on specific subsets, such as materialized or praxis components, in a more limited and less integrative manner. This gradient illustrated the variability in the scope of biocultural diversity studies and underscored the value of transdisciplinary approaches in achieving a more integrated understanding of it. Furthermore, the most comprehensive studies (Cluster 2), which integrate multiple dimensions and sub-dimensions of biocultural diversity, provide a richer understanding of the interactions between biological and cultural elements. In contrast, studies with a narrower focus may overlook key socio-ecological linkages that are essential for understanding the system and designing effective conservation strategies. This reinforces the benefits of adopting a transdisciplinary approach that integrates a wide range of biocultural components into conservation efforts. Therefore, our findings support the argument that future conservation initiatives should emphasize biocultural approaches. We suggest that research within Cluster 2 could serve as a model for future studies aiming to capture the complexity of biocultural interactions in social-ecological systems. For instance, conservationists and managers could use this type of holistic framework to understand the big picture of the system and design interventions that do not focus solely on material elements (e.g., protected species or landscape) but also integrate lived and stewardship dimensions, such as reinforcing traditional knowledge systems, supporting intergenerational transmission of cultural practices, or strengthening local governance structures for sustainable resource management.

Empirical transdisciplinary studies that encompass a comprehensive understanding of biocultural diversity and social-ecological systems can provide valuable insights for decision-making and guide sustainable culturally sensitive policies, and actions to address current complex environmental and societal challenges (Hanspach et al. 2020; Díaz-Reviriego et al. 2024). However, our in-depth analysis of the articles included in the systematic mapping revealed two persistent challenges: (1) methodological and operational challenges (e.g., lack of biocultural datasets and financial constraints) when conducting comprehensive studies, and (2) inter and transdisciplinary challenges regarding collaborative work and the inclusion of intricate cultural intangible components. Applying our classification framework could help overcome some of these barriers by offering a clearer structure to incorporate both tangible and intangible dimensions into conservation policies. In addition, from some of the analyzed papers, we learned that recent research in intangible cultural heritage (Brennan 2018; Stepputat et al. 2019) and the application of the Field Environmental Philosophy approach (Rozzi et al. 2008, 2023; Tauro et al. 2021) provide methodologies (e.g., collaborative action research, participatory mapping, collaborative ethnography, and community-based conservation actions) for navigating some of these challenges.

## Conclusion

This study aims to enhance the understanding of biocultural diversity by systematically integrating English and Spanish literature and introducing a refined classification framework that specifies components within the three main biocultural diversity dimensions proposed by Elands et al. (2019). This advancement tries to operationalize the biocultural diversity concept, offering a practical tool for more effective future inventories that could inform evidence-based decision-making for the management and conservation of socio-ecological systems. By bridging theoretical clarity with practical applicability, this work addresses a gap in existing research. Future studies should test our classification’s applicability across diverse socio-ecological contexts by conducting biocultural diversity inventories in different social-ecological systems located in various regions and countries. This will help assess the relevance of the classification and its adaptability to varying cultural and environmental conditions, ultimately contributing to a more robust and universally applicable framework.

Furthermore, our findings highlight critical geographic and thematic research gaps, particularly in Africa, Asia, and systems such as inland water, marine, and urban environments. Addressing these gaps will require targeted research toward those underrepresented regions and equitable funding for local researchers (Gavin et al. 2018). Moreover, biocultural diversity research will be required to develop more balanced approaches that consider both tangible and intangible dimensions with the same importance and apply improved methodologies to deeply study the underrepresented intangible components. Strengthening this balance is essential to fully capture the complexity of human-nature interactions and their role in fostering sustainability.

Finally, future conservation efforts should emphasize biocultural approaches (Rozzi et al. 2006) that integrate local (Shrumm and Jonas 2012) and global perspectives (Declaration of Belém 1988; UNESCO Florence Declaration 2014), empower communities through biocultural protocols, and recognize traditional knowledge and practices in policy frameworks (Cooke et al. 2022). These approaches not only safeguard biocultural diversity but also actively involve local stakeholders in co-management processes (Reed and Ceno 2015), ensuring that conservation efforts align with cultural and ecological contexts. By leveraging these tools, biocultural diversity can move beyond theoretical frameworks and become a more actively applied approach in conservation, resilience, and sustainability efforts.

## Supplementary Information

Below is the link to the electronic supplementary material.

Supplementary file1 (PDF 682 KB)

## Footnotes

1 https://www.scopus.com.

2 https://scielo.org.

3 www.redib.org.

4 www.redalyc.org.

5 https://dialnet.unirioja.es.
