# Supplementary material for: Exploring biocultural diversity: A systematic analysis and refined classification to inform decisions on conservation and sustainability
Source: Ambio. 2025 Apr 16;54(10):1581–97. doi: 10.1007/s13280-025-02168-y (PMC12405084; doi:10.1007/s13280-025-02168-y)
Supplement: Supplementary file 1 — Supplementary file1 (PDF 682 KB) [file 13280_2025_2168_MOESM1_ESM.pdf]

Exploring biocultural diversity: A systematic analysis and refined classification to inform decisions on conservation and sustainability

***** *Supplementary material may be found in the online version of the article at the publisher's website.*

AUTHOR NAMES

Irene Otamendi-Urroz [a], Cristina Quintas-Soriano [a, b], Jan Hanspach [c], Juan Miguel Requena-Mullor [a], Anna Sophie Lagies [c], Antonio J. Castro [a].

AFFILIATIONS

[a] Social-Ecological Research Laboratory, Biology and Geology Department, Andalusian Center for Global Change - Hermelindo Castro (ENGLOBA), University of Almeria, La Cañada de San Urbano, 04120, Almería, Spain. ireneota@ual.es; cristina.quintas@ual.es; juanmir@ual.es; acastro@ual.es.

[b] FRACTAL Collective, Madrid, Spain. cristina.quintas@ual.es.

[c] Faculty of Sustainability, Leuphana University Lüneburg, Lüneburg, Germany. hanspach@leuphana.de ; sophie.lagies@gmx.de.

CORRESPONDING AUTHOR ADDRESS AND EMAIL

*Correspondence: Irene Otamendi-Urroz. Social-Ecological Research Laboratory, Biology and Geology Department, Andalusian Center for Global Change - Hermelindo Castro (ENGLOBA), University of Almeria, Carretera Sacramento s/n, 04120, La Cañada de San Urbano, Almería, Spain. ireneota@ual.es. +34 636242911

SUPPLEMENTARY MATERIAL

Appendix S1. Protocol of the systematic mapping process. Private link to Figshare: https://figshare.com/s/ef7366f54896adf8f9de

Appendix S2. Database with included and excluded articles and the main reasons for the decision. Private link to Figshare: https://figshare.com/s/5477efb1f90b1e4c15a1

Appendix S3. Reviewed literature: list of references. Private link to Figshare: https://figshare.com/s/8dd9f0afa6189d5582eb

Appendix S4. Guidelines for data extraction and coding. Private link to Figshare: https://figshare.com/s/c946eace6ada40ba2d09

Appendix S5. Database with all the articles and variables coded. Private link to Figshare: https://figshare.com/s/e861f14ab4bb77e14ed8

Appendix S6. ROSES flow diagram for the systematic mapping process. The dashed lines represent the steps performed by other research groups. Private link to Figshare: https://figshare.com/s/fbc45ecd523ea4edf60b

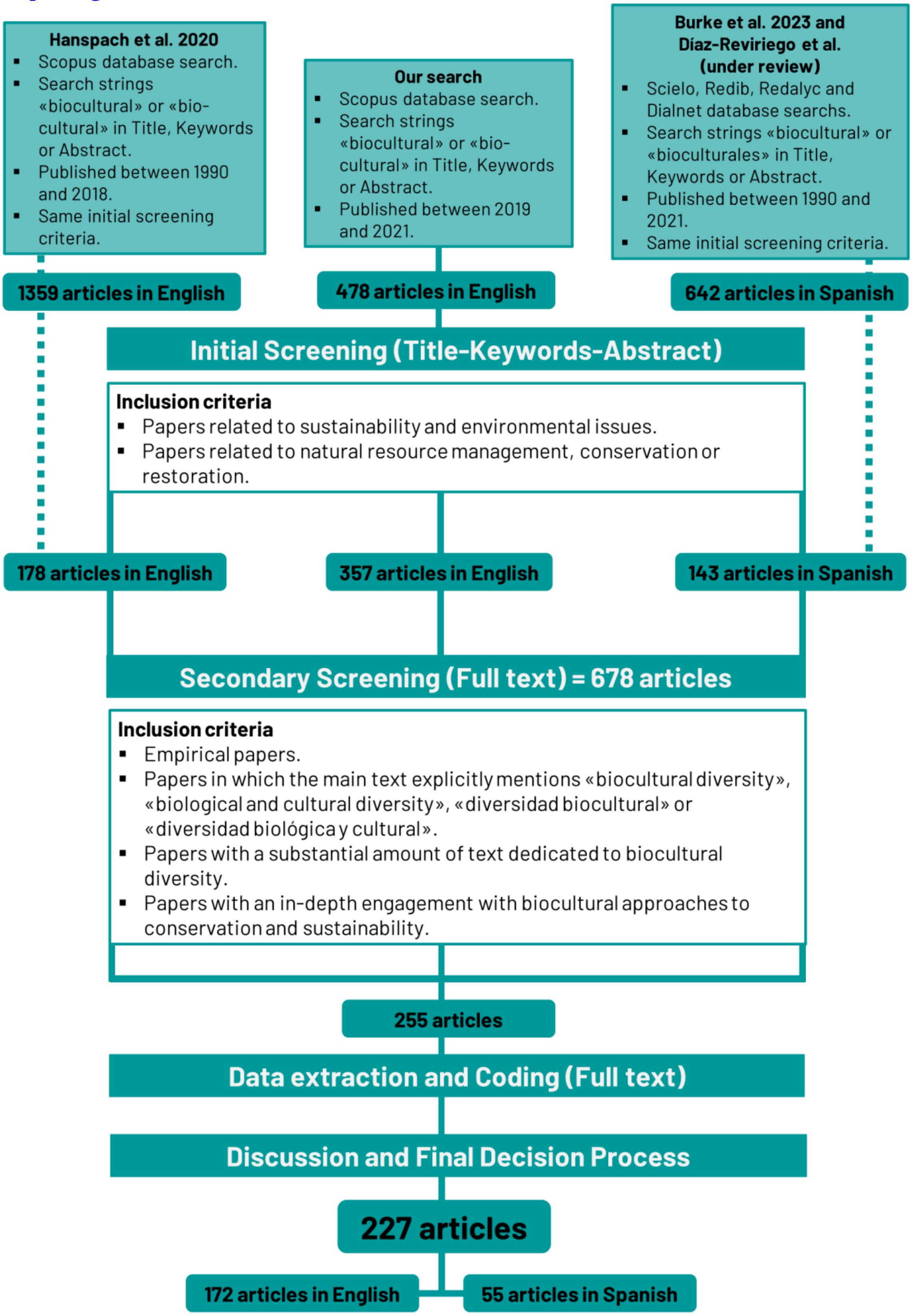

Appendix S7. Interactive map of biocultural diversity study areas. Public link to Google Maps: https://www.google.com/maps/d/edit?mid=1cCffOvdfrIoXpJteFPNGt3eWcY7U9NE&usp=sharing

Appendix S8. Cluster analysis dendrogram. Private link to Figshare: https://figshare.com/s/ec01d5a061cb61ff1de3

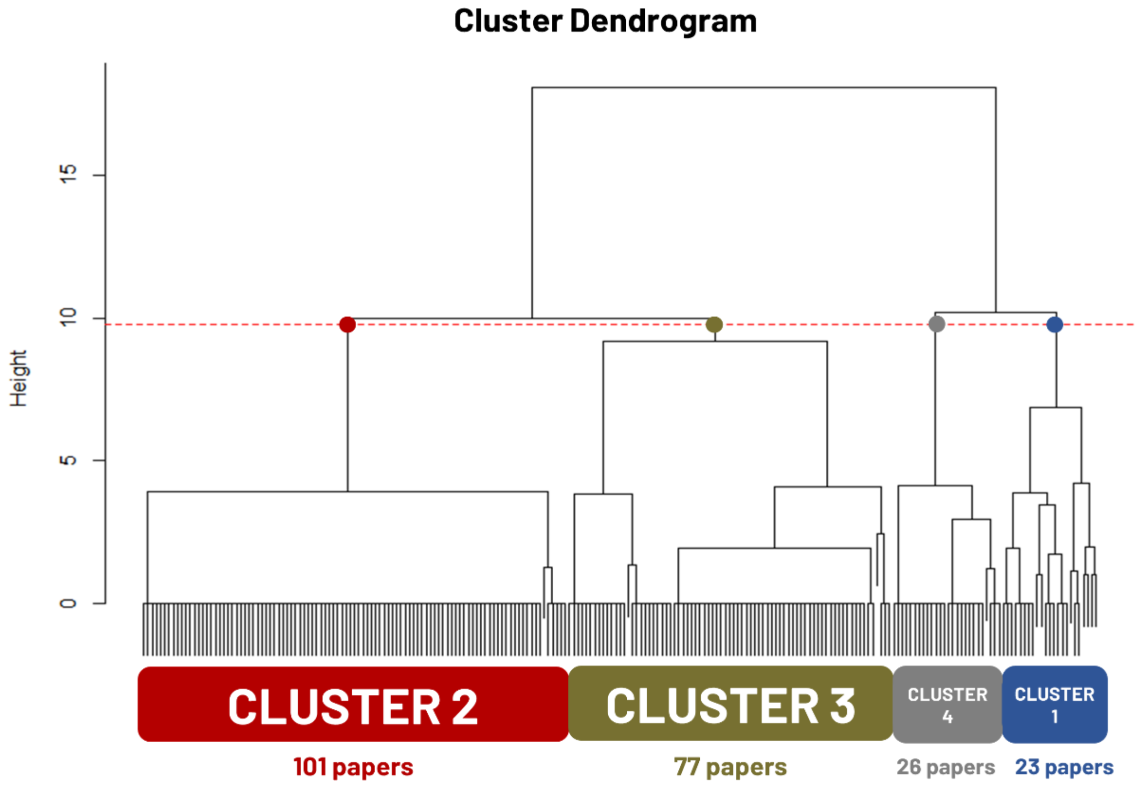

Appendix S9. Cluster analysis: list of articles included in each cluster. Private link to Figshare: https://figshare.com/s/30635174c33aa2a24510
